# Fire acupuncture for mild to moderate knee osteoarthritis: a protocol for a randomized controlled pilot trial

**DOI:** 10.1186/s13063-019-3744-2

**Published:** 2019-12-04

**Authors:** Yuan-Bo Fu, Bin Li, San-Feng Sun, Hui-Lin Liu, Xin Wang, Shao-Song Wang, Fan Zhang, Xin Du, Du-Juan Ge, Lei Shang, Rui-Li Liang, Li-Na Wang, Fang Yuan, Jing-Qing Sun, Jun-Wei Chen

**Affiliations:** 1 The Department of Acupuncture and Moxibustion, Beijing Hospital of Traditional Chinese Medicine, Capital Medical University, Beijing Key Laboratory of Acupuncture Neuromodulation, No. 23 Meishuguanhou Street, Dongcheng District, Beijing, 100010 China; 2 Beijing Huairou District Hospital of Traditional Chinese Medicine, No. 1 Houheng Street, Huairou District, Beijing, 101400 China; 3 Beijing University of Chinese Medicine, No. 11, Bei San Huan Dong Lu, Chaoyang District, Beijing, 100029 China

**Keywords:** Knee Osteoarthritis, Fire Acupuncture, Basic health management

## Abstract

**Background:**

Knee osteoarthritis (KOA) is one of the most common bone and joint diseases. As one of the main non-drug therapies, acupuncture is widely used to treat KOA, although the evidence for its efficacy is inconclusive. The objective of this pilot trial is to clarify the clinical efficacy and safety of fire acupuncture in the treatment of mild to moderate KOA and to provide high-quality data for further research.

**Methods/design:**

This study is a prospective randomized controlled pilot trial in which 120 patients with mild to moderate KOA will be randomly allocated in equal proportions to a fire acupuncture group or a general acupuncture group. They will receive acupuncture for six sessions over 2 weeks. The primary end point is success rate, which will be calculated based on the change from baseline of the pain and function scores in the Western Ontario and McMaster Universities Osteoarthritis Index at 4 weeks. Secondary end points include the proportion of patients achieving clinical improvement based on: (1) the OMERACT-OARSI responder criteria, (2) levels of matrix metalloproteinase 3, interleukin 1β, and tumor necrosis factor α in blood, and (3) a subjective efficacy evaluation from patients.

**Trial registration:**

Chinese Clinical Trial Registry, ChiCTR1800019162. Registered on 29 October 2018.

## Background

Knee osteoarthritis (KOA) is one of the most common musculoskeletal disorders [1]. Its prevalence rate is 49% among people over 60 and 80% among people over 75 in China. One third of people over 65 suffer from KOA, according to epidemiological surveys [2]. Worldwide, an estimated 25,000 people suffer from KOA [3]. Most have radiological or clinical evidence of osteoarthritis [4], and the incidence of the disease has been increasing with the aging of the population [5]. Its high incidence and high disability rate not only bring a heavy economic burden to patients, but also seriously affect their quality of life [6]. If symptoms persist, patients may have low levels of social activity and abnormal psychological functioning, which may eventually lead to a decline in the quality of life and even an inability to live independently, causing heavy psychological pressure and a burden on the patient and their family.

Analgesics—such as non-steroidal anti-inflammatory drugs, opioids, and non-opioid central analgesics (such as tramadol)—are still recommended to treat the disease, according to the guidelines for the diagnosis and treatment of KOA published by the American Academy of Orthopaedic Surgeons in 2013 [7] and those published by the National Institute for Health and Care Excellence in 2014 [8]. However, drug treatments can cause serious gastrointestinal complications, such as ulcers, perforations, and bleeding, and the risk increases with age and treatment duration. At present, the treatment for KOA is pain relief, symptomatic treatment, maintenance, or improvement of joint function, since there is no effective way to completely block or reverse the development of KOA. Most patients with advanced KOA have to “cure” it by undergoing knee replacement. Unfortunately, many KOA patients are unable to undergo a knee replacement due to their physical condition or affordability. Therefore, a safe and effective therapy for KOA that is free of side effects is urgently required to improve patients’ quality of life and reduce the national medical burden.

Acupuncture is important in traditional Chinese medicine as a non-drug therapy. It has been used for more than 2000 years in China [9]. In fire acupuncture (FA), a heated needle is inserted into an acupuncture point to eliminate the symptoms of a disease [10]. A meta-analysis published in *Evidence-Based Complementary and Alternative Medicine* showed that FA has a higher response rate and higher cure rate in the treatment of KOA [11].

Although FA is widely used to treat KOA and has achieved good results, there is still a lack of good medical evidence to verify whether it is beneficial. In addition, the molecular biological mechanism is still unclear. Fire needle therapy has been developed over thousands of years. It has a wide range of clinical applications in China, with a definite curative effect and a wide range of indications. Studies have shown that FA can reduce the level of some inflammatory cytokines that are elevated in KOA, such as interleukin 1 (IL-1), interleukin 6 (IL-6), and tumor necrosis factor α (TNF-α) [12].

Nevertheless, none of the 13 studies included in the meta-analysis in *Evidence-Based Complementary and Alternative Medicine* [11] were of sufficient quality. Most of the research into FA quoted in the literature is of poor quality, for example, the randomization methodology is often unclear and there can be issues with the selection of the control group. Thus, doctors and patients may have concerns about the efficacy and safety of FA. Therefore, high-quality prospective randomized controlled trials are needed to determine the clinical efficacy and safety of FA in treating mild and moderate KOA and to explore the mechanisms of action.

## Methods/design

### Study design

**Fig. 1:**
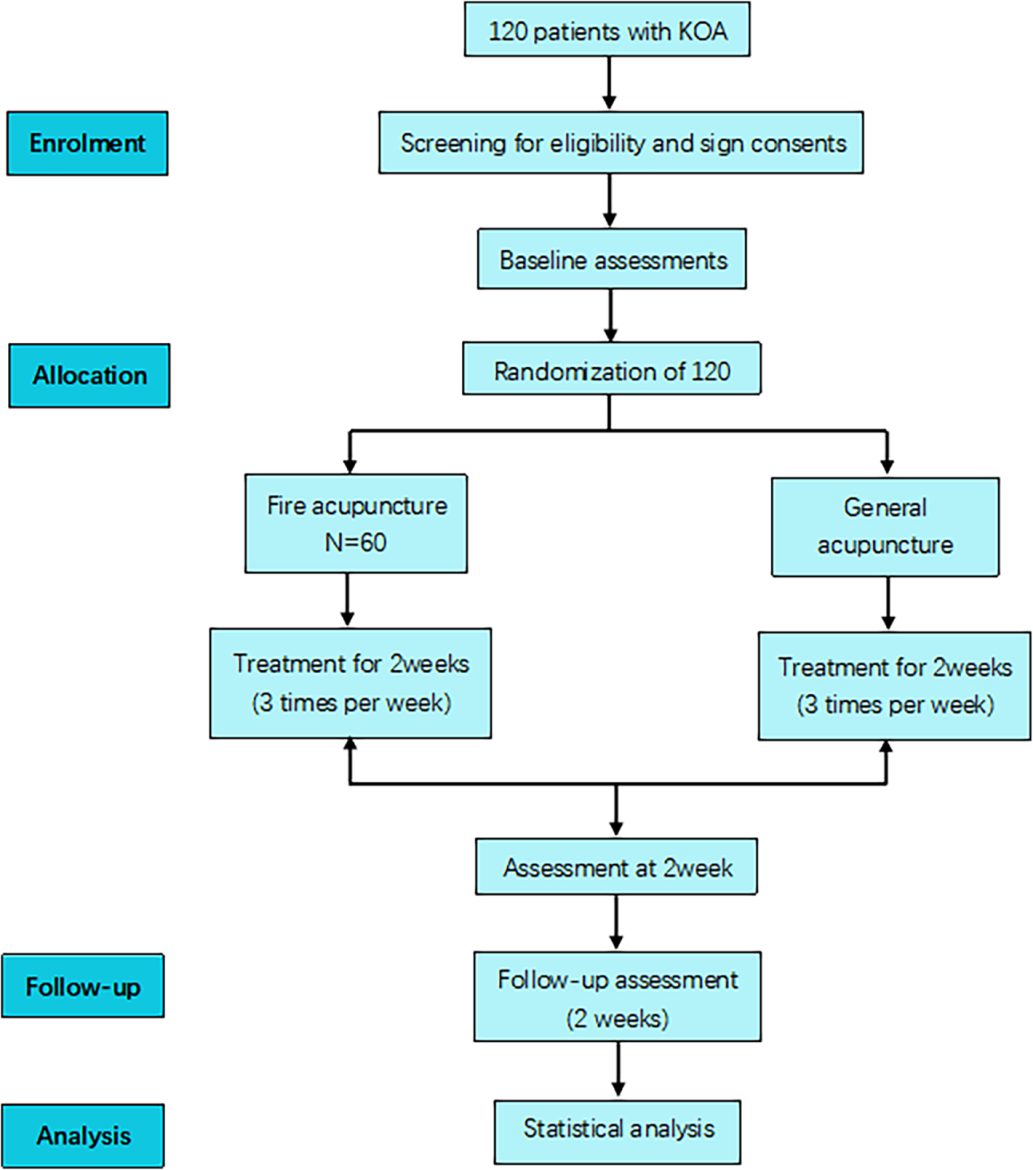
Trial flow chart. KOA, knee osteoarthritis

The study is a prospective multi-center parallel randomized controlled trial. We will enroll patients from Beijing Hospital of Traditional Chinese Medicine (Capital Medical University) and Beijing Huairou District Hospital of Traditional Chinese Medicine with mild to moderate KOA, according to the inclusion and exclusion criteria, from January 2019 to December 2019. Participants will be randomly allocated to one of two groups and will receive treatment for 2 weeks. The trial has been registered with the Chinese Clinical Trial Registry (ChiCTR1800019162). All items from the World Health Organization’s trial registration data set have been entered into the registry. The intervention includes six sessions of acupuncture and two follow-up visits (Fig. 1 and Table 1).

**Table 1:** Schedule of enrollment, interventions, and assessments

	Baseline	Treatment phase	Follow-up phase	
	−1 day	0 day	2 weeks	4 weeks
Patients				
Informed consent	×			
Sign informed consent		×		
Medical history	×			
Physical examination	×			
Randomization		×		
Intervention FA group (*n* = 60)	Six sessions of FA			
Comparisons GA group (*n* = 60)	Six sessions of GA			
Outcomes				
WOMAC		×	×	×
OMERACT–OARSI		×	×	×
MMP-3		×	×	
IL-1β		×	×	
TNF-α		×	×	
SEE		×	×	×
Participant safety				
Adverse events		×	×	×

*FA* fire acupuncture, *GA* general acupuncture, *IL* interleukin, *MMP-3* matrix metalloproteinase 3, *SEE* subjective efficacy evaluation, *TNF-α* tumor necrosis factor α, *WOMAC* Western Ontario and McMaster Universities Osteoarthritis Index

### Inclusion criteria

To be eligible, patients must meet all of the following inclusion criteria:

Aged 38–85 years (either sex)

Meet the diagnostic criteria for primary KOA

Mild to moderate KOA (Kellgren–Lawrence score 1–2)

Able to receive FA treatment

Voluntary participation in the study

Able and willing to sign the informed consent form

### Exclusion criteria

To be eligible, patients must not meet any of the following inclusion criteria:

1. Secondary KOA
2. The knee joint has a history of trauma or surgery in the past year
3. Severe joint deformity (varus or valgus ≥8°)
4. Injection of glucocorticoid into the articular cavity of the target knee within 3 months
5. Injection of sodium hyaluronate into the articular cavity of the target knee within 2 weeks
6. Have received acupuncture or moxibustion for symptomatic hip arthritis or patellar arthritis on the same side of the target knee joint within 2 weeks
7. Symptomatic hip or patellar arthritis on the same side of the target knee joint
8. Inflammatory or other rheumatic disease
9. Acute knee meniscus injury
10. Acute knee injury or fracture around the knee
11. Tumor around the knee
12. Tuberculosis or idiopathic osteonecrosis
13. Taking paracetamol, non-steroidal anti-inflammatory drugs, or analgesics for other diseases
14. Uncontrolled hypertension or diabetes
15. Severe cardiovascular, pulmonary, liver, spleen, kidney, or hematopoietic disease
16. Tumor hemorrhages
17. Mental illness
18. Scar diathesis or skin sensory impairment
19. Pregnant or lactating women

### Randomization and allocation concealment

Random permuted blocks will be used. The allocation to the FA group and to the general acupuncture (GA) group will be in a 1:1 ratio. Opaque numbered envelopes will be used to conceal the allocation. Researchers will open the envelopes according to the order of enrollment. The acupuncturists will not be involved in the randomization. Data collectors, data evaluators, and statisticians will be blinded to group assignment. Participants will not be blinded.

### Interventions

This study conforms to the CONSORT [13] and STRICTA [14] criteria. All acupuncturists are required to have had Chinese medical practitioner licenses for at least 20 years and they will receive the same training under the clinical treatment program prior to the study.

The acupuncture sessions will be for 30 min. There will be three sessions per week for 2 weeks, giving six sessions in total. Sterilized medium and coarse He’s fire needles will be used for the FA group. Disposable, sterile steel, 0.30 × 50 mm acupuncture needles (Ande disposable acupuncture needles, Guizhou Medical Co., Guizhou, China) will be used for the GA group. If participants experience pain of intensity ≥ 4 on a 10 cm visual analog scale [15], they will be given Celebrex. Adverse reactions during treatment will be recorded in the case report form.

Patients in both groups will receive basic health management. Blood tests will be performed on day 0 and the second week after the six acupuncture sessions. In this study, we strengthened patients’ self-management by giving them a detailed manual on managing KOA. The manual was developed by the research team based on the second edition of the evidence-based medical guidelines for KOA published by the American Academy of Orthopaedic Surgeons. The manual explains the natural course of the disease, gives advice on how to avoid psychological effects, and describes the drugs used and their side effects. The manual recommends that they (1) maintain good posture, (2) avoid being stationary for a long time, (3) do not run or jump, and (4) minimize their use of stairs, etc. The contents of the manual are explained to the patient in detail before randomization. Subjects were asked to comply with these self-management recommendations for at least 4 weeks after being randomized. Patients with severe pain may take non-steroidal anti-inflammatory drugs, including topical drugs and systemic drugs. They are asked to record the drug used, the frequency of administration, the dosage, and any adverse drug reactions in the manual.

### FA group

**Fig. 2:**
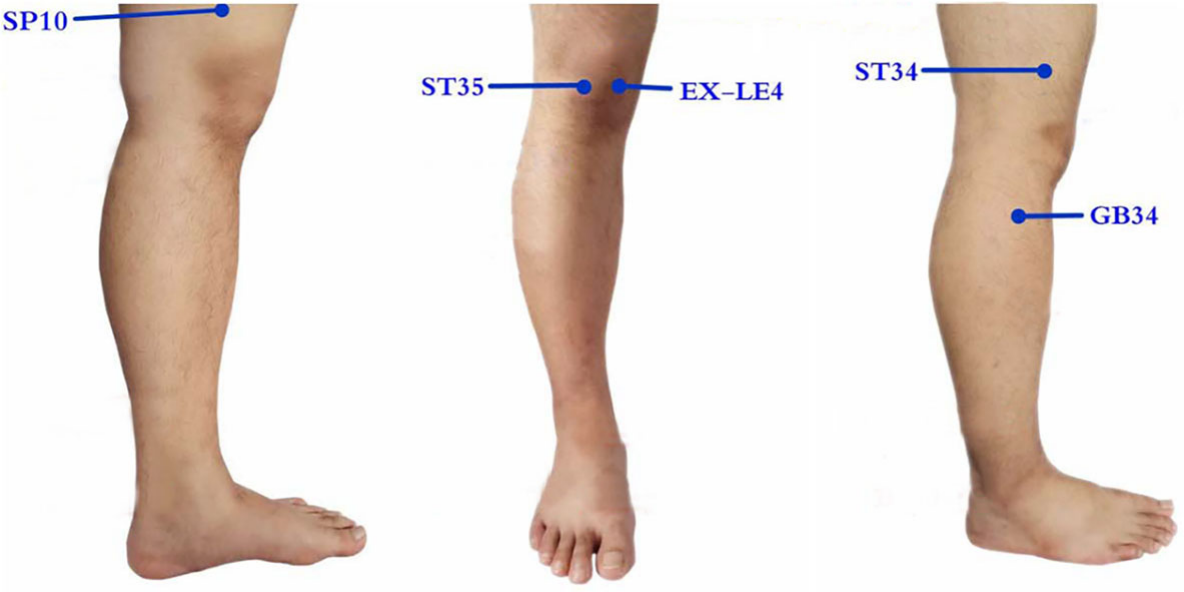
Acupuncture points for both groups

Acupuncture points are selected based on the *bi* syndrome in traditional Chinese medicine. There are five local points (ST34, ST35, SP10, EX-LE4, and GB34) and one or two pain points (*ashi* points) (Fig. 2). The procedure is as follows:

1. Place the patient in a supine position with their knees bent.
2. Disinfect the acupuncture points.
3. Heat the tip and middle of the acupuncture needle until it glows red
4. Pierce each point three times to a depth of about 0.5 cm, moving the needle quickly in and out. The needle will be heated before each insertion.
5. Press the acupuncture eyes for a moment with a sterile dry cotton ball.

### GA group

2The acupuncture points used are the same as those for the FA group. The procedure is as follows:

1. Place the patient in a supine position with their knees bent.
2. Disinfect the acupuncture points.
3. Insert a needle into each point to the optimum depth. The needles remain in place for approximately 30 min.
4. Remove all needles and use sterile cotton balls to avoid bleeding.

Acupuncturists are instructed to achieve *de qi* and to stimulate the needles manually for at least 10 s.

### Primary outcome

The success rate will be calculated based on the change from the baseline in the total score for the Western Ontario and McMaster Universities Osteoarthritis Index (WOMAC) [16, 17] at weeks 2 and 4, which has five dimensions: (1) pain, (2) joint stiffness, (3) joint physiology, (4) social functioning, and (5) emotion, with a total score of 24. Higher scores represent a worse condition.

### Secondary outcomes

**Fig. 3:**
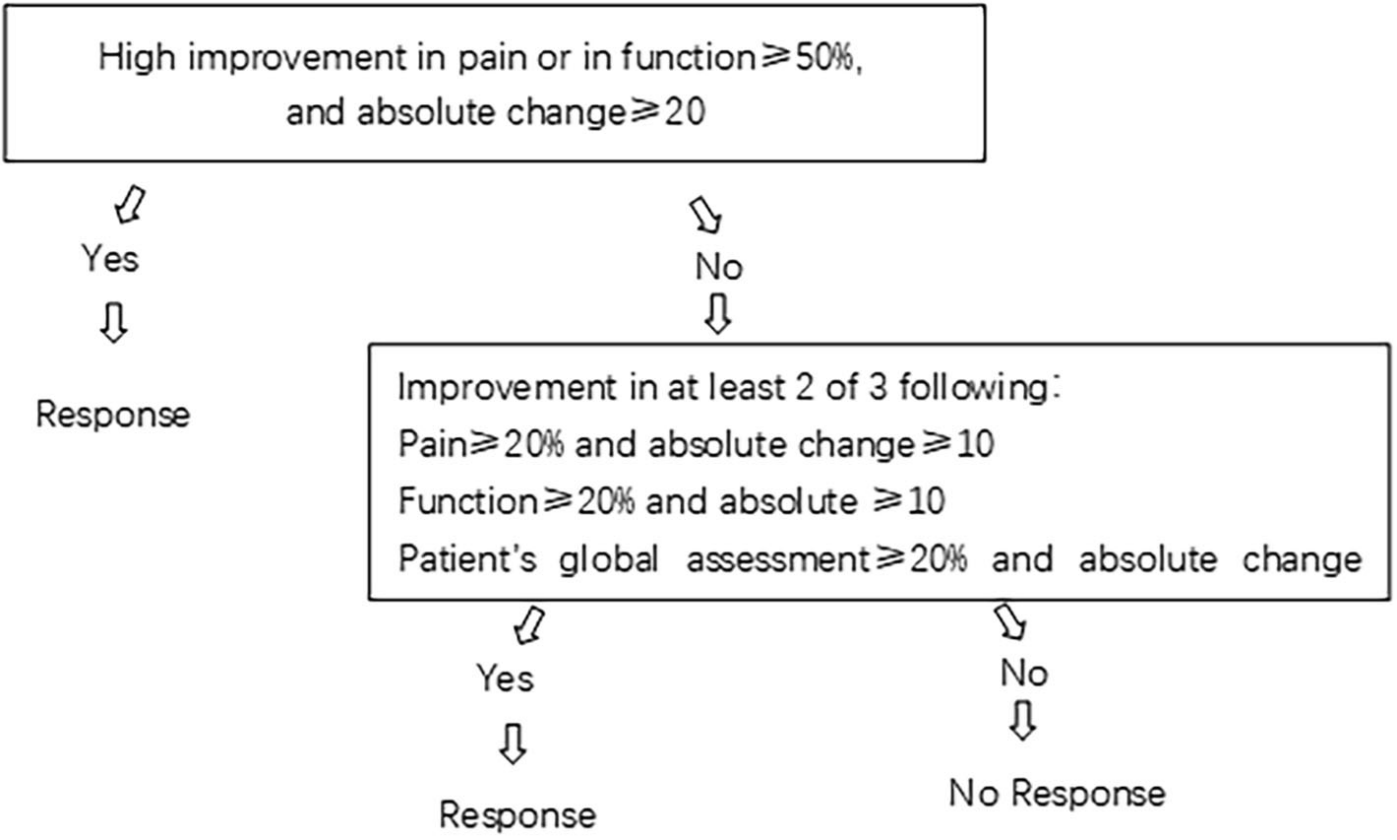
OMERACT–OARSI responder criteria

1. The proportion of patients with a clinical improvement: The OMERACT-OARSI responder criteria will be used at weeks 2 and 4 to assess whether a patient has a clinical improvement, and compare whether there is a difference in improvement between the two groups (Fig. 3).
2. Subjective efficacy evaluation: At weeks 2 and 4, a research assistant will ask each patient about the effects of the therapy they received on their KOA compared to the baseline. The evaluation is scored as follows: greatly aggravated –3, moderately aggravated −2, mildly aggravated −1, totally unhelpful 0, very little help 1, moderate assistance 2, and great help 3.
3. Laboratory indicators: Patients’ peripheral blood will be collected at weeks 0 and 2, and levels of matrix metalloproteinase 3 (MMP-3), IL-1β, and TNF-α will be measured by an enzyme-linked immunosorbent assay.

### Sample size

Since no previous multicenter randomized controlled trials have studied the efficacy of FA in treating KOA, this study will provide effect size data for sample size calculations in subsequent large-scale randomized controlled trials. Overall, 120 outpatients and inpatients will be recruited at the two research centers, giving 60 participants per group.

### Statistical analysis

This project will use a remote data capture system. Data will be double entered by two clerks. Once locked, the SAS database will be delivered to the statisticians along with a description of the data structure and a coding table. The statistical analysis will be performed by statisticians who are blinded to the allocation and are not involved in other aspects of the study.

All randomized subjects will be analyzed statistically according to the intention-to-treat principle. The last observation carried forward method will be used to impute missing values. The software SPSS (V.18.0 KO for Windows) will be used to analyze the results. All statistical tests will be two sided, and *p* < 0.05 will be considered statistically significant. Continuous data will be described by mean ± standard deviation, median, maximum, and minimum. Discrete data will be described by frequencies and percentages. Continuous data will be analyzed by a paired *t*-test (if normally distributed) or a non-parametric test (if not normally distributed). Discrete data will be analyzed with a Pearson chi-squared test or a rank sum test.

The cartilage exfoliation rate due to adverse events will be determined for each center and for each group. The exfoliation rates will be analyzed with Fisher’s exact test. Using the case report forms, we will evaluate whether each participant received the prescribed dose and number of treatments according to the research plan.

An effectiveness analysis will be conducted using the randomized populations. Participants who have not received any treatment and participants who have received treatment but for whom there is no valid evaluation data will be considered as missing and will be included in the effectiveness analysis.Participants who cannot tolerate treatment after the start of observation, have serious side effects, have less than 2 weeks of treatment, or are lost to follow-up will be considered as cases of shedding. When the participant falls off, the investigator should make an appointment for follow-up, telephone or letter to contact the participant as much as possible, ask the reason, and record the last treatment time to complete the evaluation project. For trial cases withdrawn due to adverse reactions and ineffective treatment, the investigator should take corresponding treatment measures according to the actual situation of the subject. The investigator should indicate the main reason for terminating the trial. If the exfoliation rate exceeds 20% of the total number of participants, the study will be considered a failure and terminated.

### Quality supervision and management

To improve patient compliance with the treatment and health management plans, we propose to use the patient manuals, regular mobile phone reminders and other methods, and patients will be compensated financially. The data monitoring committee (DMC) consists of clinical experts, statisticians, experimenters, third-party inspectors, etc. The DMC will analyze summarized data by the third-party blind method to ensure the authenticity and reliability of the research results. The DMC will monitor the quality of the study. It is independent of the sponsors and there are no competing interests. Specialized institutions designated by the funders will conduct a test acceptance and funding audit.

The clinical trial will be suspended or discontinued if the safety of any participant is compromised or if any serious adverse reactions are not reported within the prescribed time. If the trial is suspended or discontinued, the participants, the project funder (Beijing Huairou District Science and Technology Committee), and the ethics committee will be informed of the reason.

### Patient and public involvement

We will make details of the study available to the public through online publicity and posters. Interested patients can visit our research center before participating in the study to learn more about it. Patients will not be involved in the design of the study or recruitment.

### Ethics and dissemination

#### Patient consent and dissemination policy

This protocol has been registered with the Chinese Clinical Trial Registry. All participants will be given ample time to consider whether to participate in the study and to sign the informed consent, which includes consent for blood tests. All blood samples taken from participants will be destroyed after the trial. Participants can withdraw their informed consent at any time during the study. We will publish the results of the trial in a peer-reviewed clinical journal for wide dissemination. Participants who experience severe adverse reactions or irreversible injuries as a result of the trial will be reasonably compensated, according to the circumstances.

#### Access to data and confidentiality

Only research team members have access to the research data. All data will be kept strictly confidential during the study. On completion of the study, participants will receive a personal summary of the data collected in the study.

## Discussion

KOA is a common disease with a high disability rate. It results in significant financial losses and is a burden for patients and society. At present, the treatment of KOA is still at the level of pain relief and symptomatic treatment, and the etiology and pathogenesis of KOA are still unclear [18]. Studies have shown that the main pathological changes of KOA are the degeneration of the articular cartilage and a change in synovial composition [19].

As a major non-drug therapy, acupuncture is widely used to treat KOA. Studies have shown that acupuncture can effectively relieve pain and improve the mobility of the knee [11]. FA is a combination of acupuncture and heat. It has a good curative effect on musculoskeletal diseases by warming channels to clear collaterals and veins. Studies have shown that FA can reduce the level of some inflammatory cytokines that are elevated in KOA [12]. Therefore, we suspect that KOA may be related to changes in some inflammatory factors.

Compared with other clinical treatments, FA has the advantages of significant efficacy and high safety, and moreover, it is readily accepted for the treatment of KOA by patients. Although a published meta-analysis [10] showed that FA has advantages in KOA treatment, high-quality randomized controlled trials investigating FA as well as research based on disease classification or with stratification are still lacking. Therefore, this prospective randomized parallel controlled trial is necessary to assess the safety and efficacy of FA in treating KOA and to indicate the mechanisms of action.

A carefully designed clinical trial has a suitable control group. We know from the literature review as well as our own clinical experience that GA is also effective for KOA. It is also a relatively safe non-pharmacological treatment that is available in most countries. Thus, we used GA for the control group rather than another treatment that would not be as effective. In this study, the participants in both groups will receive three acupuncture sessions a week for 2 weeks, for a total of six sessions.

The target disease of this study is mild and moderate KOA. Therefore, if this study proves that FA is effective in treating KOA, the next step is to evaluate the efficacy of FA in treating severe KOA. In addition, this study is an initial exploration of the correlation between FA and inflammatory factors in peripheral blood. If this correlation is verified, then the mechanism for how FA affects KOA can be further explored from the perspective of molecular biology (Additional file 1).

### Strengths

In principle, FA has been recognized in China as a relatively safe non-drug treatment. This study will provide further high-quality clinical evidence on the safety of FA. The planned study has the potential to demonstrate that FA is a novel, practical, and effective treatment for KOA. If FA is effective in alleviating pain and improving joint function in patients with KOA, then it could be adopted as a cheap non-drug therapy instead of potentially addictive drugs or surgery, which may help in reducing the living and economic burden on patients. Since FA is simple, fast, cheap, and easy to implement, then if its efficacy and safety in treating KOA are recognized, doctors will have more treatment options for KOA.

### Limitations

Acupuncturists and participants will not be blinded due to the nature of the intervention. Participants can not be blinded to the study design due to the feeling of the skin during treatment. The acupuncturists will not be involved in assessing outcomes or the data analysis.

Although the duration of treatment for each participant is only 4 weeks, it is possible to compare the efficacy and short-term effect of FA and GA. Due to the short observation time, however, it will not be possible to determine the long-term efficacy of using fire needles. Levels of the inflammatory factors in peripheral blood may increase during the follow-up period rather than decrease as we expect.

### Trial status

This trial is currently recruiting participants (protocol version 2.0, 20 April 2019). The study will run from 1 June 2019 to 31 March 2020.

## Supplementary information

**Additional file 1.** SPIRIT 2013 checklist.

## Data Availability

The original data will be released within 6 months of completion of the trial. The specific disclosure method for the original data will be selected according to the research process.

## Abbreviations

DMC: Data monitoring committee
FA: Fire acupuncture
GA: General acupuncture
IL: Interleukin
KOA: Knee osteoarthritis
MMP-3: Matrix metalloproteinase 3
SEE: Subjective efficacy evaluation
TNF-α: Tumor necrosis factor α
WOMAC: Western Ontario and McMaster Universities Osteoarthritis Index

## References

[1] Hochberg MC, Altman RD, April KT (2012). American College of Rheumatology 2012 recommendations for the use of nonpharmacologic and pharmacologic therapies in osteoarthritis of the hand, hip, and knee. Arthritis Care Res (Hoboken).

[2] Mavrommatis CI, Argyra E, Vadalouka A (2012). Acupuncture as an adjunctive therapy to pharmacological treatment in patients with chronic pain due to osteoarthritis of the knee: a 3-armed, randomized, placebo-controlled trial. Pain.

[3] O'Neill TW, McCabe PS, McBeth J (2018). Update on the epidemiology, risk factors and disease outcomes of osteoarthritis. Best Pract Res Clin Rheumatol.

[4] Goldring MB, Goldring SR (2007). Osteoarthritis. J Cell Physiol.

[5] Baker K, McAlindon T (2000). Exercise for knee osteoarthritis. Curr Opin Rheumatol.

[6] Higgs R (2010). Osteoarthritis: Concentrated efforts to detect early OA. Nat Rev Rheumatol.

[7] Brown GA (2013). AAOS clinical practice guideline: treatment of osteoarthritis of the knee: evidence-based guideline, 2nd edition. J Am Acad Orthop Surg.

[8] National Clinical Guideline Centre. Osteoarthritis: Care and Management in Adults. London; 2014. https://www.ncbi.nlm.nih.gov/pubmed/25340227.

[9] Wu JN (1996). A short history of acupuncture. J Altern Complement Med.

[10] China National Standardization Administration SAoQS, Inspection and Quarantine (2009). Standardized manipulations of acupuncture and moxibustion-Part 12:Fire acupuncture.

[11] Wang Y, Xie X, Zhu X (2016). Fire-needle moxibustion for the treatment of knee osteoarthritis: a meta-analysis. Evidence-Based Complement Alternat Med.

[12] Hu QS, Zhang QR, Jia CS, et al. Clinical randomized controlled observation of the treatment of senile knee osteoarthritis with needle acupuncture and its effect on inflammatory cytokines in knee joint cavity. Acupuncture Research. 2011;(02):110-5. 21717778

[13] Schulz KF, Altman DG, Moher D (2010). CONSORT 2010 statement: updated guidelines for reporting parallel group randomised trials. BMJ.

[14] MacPherson H, Altman DG, Hammerschlag R (2010). Revised Standards for Reporting Interventions in Clinical Trials of Acupuncture (STRICTA): extending the CONSORT statement. PLoS Med.

[15] Liu CZ, Xie JP, Wang LP (2014). A randomized controlled trial of single point acupuncture in primary dysmenorrhea. Pain Med.

[16] Bellamy N, Buchanan WW, Goldsmith CH (1988). Validation study of WOMAC: a health status instrument for measuring clinically important patient relevant outcomes to antirheumatic drug therapy in patients with osteoarthritis of the hip or knee. J Rheumatol.

[17] Goldsmith CH, Boers M, Bombardier C (1993). Criteria for clinically important changes in outcomes: development, scoring and evaluation of rheumatoid arthritis patient and trial profiles. OMERACT Committee. J Rheumatol.

[18] Gobbi A, Lad D, Karnatzikos G (2015). The effects of repeated intra-articular PRP injections on clinical outcomes of early osteoarthritis of the knee. Knee Surg Sports Traumatol Arthrosc.

[19] Aigner T, Kim HA (2002). Apoptosis and cellular vitality: issues in osteoarthritic cartilage degeneration. Arthritis Rheum.

